# The impact of aging on cardiac repair and regeneration

**DOI:** 10.1016/j.jbc.2024.107682

**Published:** 2024-08-17

**Authors:** Iqra Anwar, Xinghua Wang, Richard E. Pratt, Victor J. Dzau, Conrad P. Hodgkinson

**Affiliations:** Mandel Center for Heart and Vascular Research, Duke Cardiovascular Research Center, Duke University Medical Center, Durham, North Carolina, USA

**Keywords:** aging, regeneration, heart, cardiac, fibroblast, cardiomyocyte, mitochondria

## Abstract

In contrast to neonates and lower organisms, the adult mammalian heart lacks any capacity to regenerate following injury. The vast majority of our understanding of cardiac regeneration is based on research in young animals. Research in aged individuals is rare. This is unfortunate as aging induces many changes in the heart. The first part of this review covers the main technologies being pursued in the cardiac regeneration field and how they are impacted by the aging processes. The second part of the review covers the significant amount of aging-related research that could be used to aid cardiac regeneration. Finally, a perspective is provided to suggest how cardiac regenerative technologies can be improved by addressing aging-related effects.

The inability of the adult human heart to repair itself makes it particularly vulnerable to the effects of aging. Cardiac aging results in the permanent loss of cardiac muscle cells, mitochondrial dysfunction, and development of fibrosis. These changes in the heart are progressive; they negatively impact cardiac function and are fertile ground for heart failure. In addition, aging also weakens the response of the heart to cardiac injury. Thus, in the aged, cardiac injury is often associated with poorer outcomes. The field of cardiac regeneration seeks to reverse fibrosis, induce vascularization, and replenish the cardiac muscle cell population. These would be useful to reverse the effects of aging. However, seeing as most of this research has been conducted in young animals, the relevance to older animals is often unclear. This review discusses attempts to regenerate the damaged heart as well as the impact of aging thereon. The review concludes with a discussion of how research into the mechanisms of cardiac regeneration can be repositioned to reverse the deleterious effects of cardiac aging.

## Aging changes the heart’s response to cardiac injury

As the heart ages, the capacity of the organ to respond to increased workload decreases. The stress this entails promotes diastolic dysfunction, arrhythmias, and heart failure (1). These changes to the normal function of the heart ensure that young and aged adults respond differently to cardiac injury.

In young adults, cardiac injury initially induces an inflammatory response, whereby neutrophils, M1 macrophages, T cells, and B-cells invade the injury area to remove dead cells and initiate the reparative phase. The reparative phase is associated with a shift in immune cell populations which actively resolve inflammation and induce fibroblasts to secrete fibrous tissue proteins. The latter results in a stable scar (2). In contrast, the inflammatory response in older hearts following injury is muted, resulting in delayed clearance of dead cells. Moreover, fibroblast responses to fibrotic cues are markedly weaker. Fibrous protein production is dampened, leading to weaker and less stable scars. Weaker scars affect cardiac function by increasing systolic dysfunction and dilative remodeling. Dampened immune and fibroblast responses in aged individuals lower the capacity to respond to stressors (2).

## The effect of aging on molecular mechanisms for cardiac regeneration

**Figure 1:**
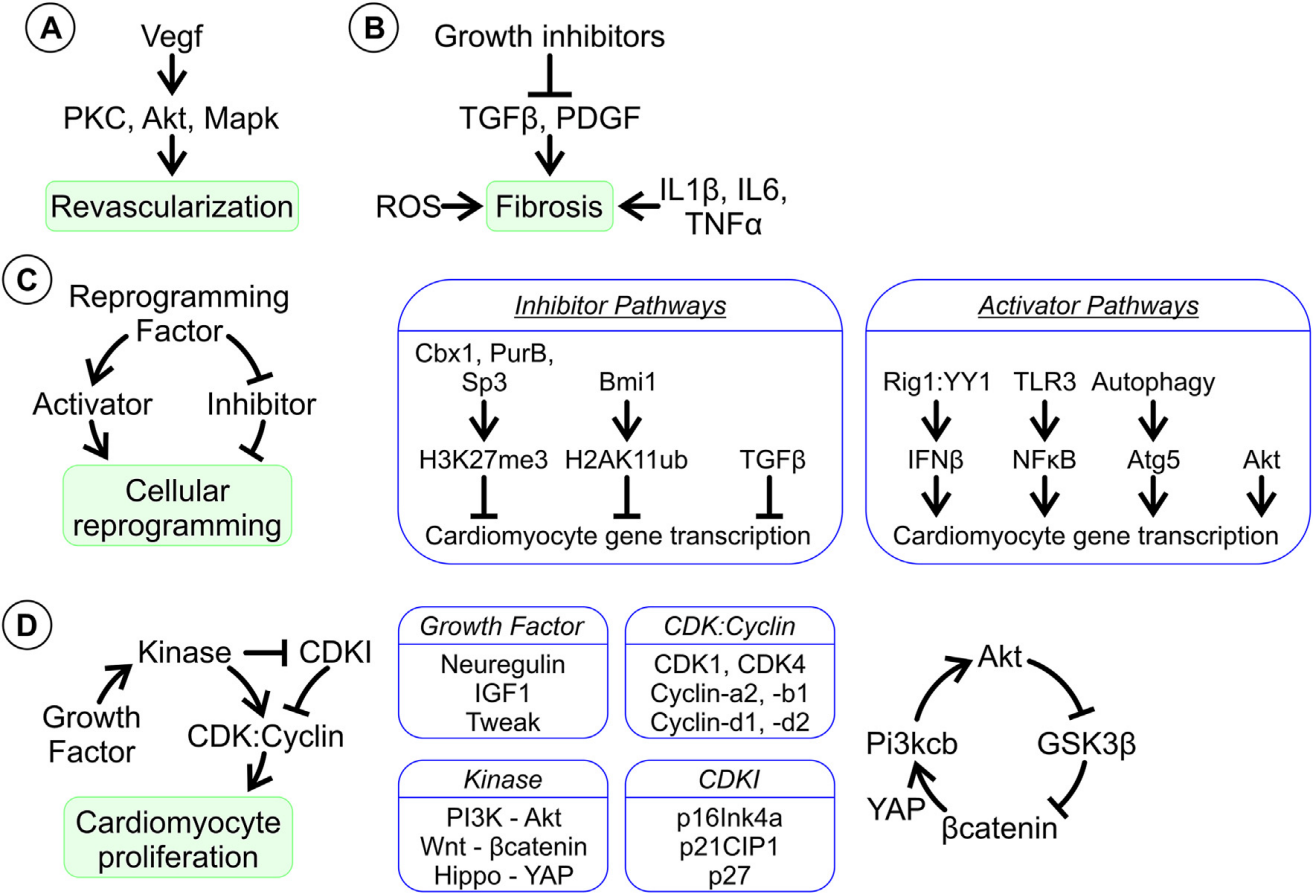
**Mechanisms in mammalian cardiac regeneration.***A*, VEGF therapies for revascularization are undergoing clinical evaluation for safety. The molecular mechanism involves the indicated kinases. *B*, triggers for fibrosis include ROS, proinflammatory cytokines such as IL6, and growth factors including TGFβ. TGFβ inhibitors are showing promise in preclinical animal models. *C*, cellular reprogramming refers to the process, whereby scar fibroblasts are converted *in situ* into new cardiomyocytes. Ostensibly, reprogramming factors work by modulating the activity/function of activators and inhibitors. Inhibitor pathways are mainly epigenetic, such as H3K27me3 and H2AK11ub. These epigenetic motifs are regulated by the indicated proteins. These proteins are direct targets for reprogramming factors. Less is known about TGFβ inhibition. Various activator pathways have been reported including immunity (Rig1, TLR3, and IFNβ), autophagy (Atg5), and growth factor signaling (Akt). *D*, cardiomyocyte proliferation is nonexistent in the adult mammalian heart. Proliferation can be induced through certain growth factors or the overexpression of various CDKs and their cognate cyclins. Another approach is targeting cardiomyocyte cell cycle inhibitors. Manipulation of various kinase signaling pathways has also proven fruitful. Interestingly, these kinase pathways appear to augment each other’s activity. CDK, cyclin-dependent kinase; ROS, reactive oxygen species; TLR, toll-like receptor; VEGF, vascular endothelial growth factor.

Cardiac regeneration research has predominantly focused on angiogenesis, reducing fibrosis, and restoring the cardiac muscle cell (cardiomyocytes) population (Fig. 1) (3). In this section, the molecular mechanisms of cardiac regeneration are discussed and how they are affected by aging.

### Angiogenesis

Blood vessels play a crucial role in delivering oxygen and metabolites to tissues while removing waste products. The formation of new blood vessels is vital for tissue regeneration. Cardiac damage provides a trigger for revascularization through arteriogenesis; whereby, existing arterioles are remodeled into larger blood vessels in an attempt to restore blood flow (4). Unfortunately, native myocardial arteriogenesis is generally insufficient. Researchers have sought ways to enhance the process by elucidating and targeting the mechanisms of vasculogenesis and angiogenesis (4). Despite a plethora of proteins and noncoding RNAs being known to regulate these processes, clinical applications have so far focused on vascular endothelial growth factor (VEGF). VEGF promotes angiogenesis by stimulating the kinases PKC, PI3K, Akt, and MAPK (5, 6, 7) (Fig. 1*A*). Various VEGF variants exist (*e.g.*, VEGF-A and VEGF-B) and one of the most advanced therapeutics for revascularization is an mRNA that produces VEGF-A (8, 9, 10, 11). Unfortunately, clinical trials have not yielded particularly encouraging results.

Compounding these issues, angiogenesis is likely to be more difficult in aged individuals. Vascular progenitors decrease significantly in number with age (12). Moreover, studies in rodents show that aging impairs angiogenesis in response to injury by inhibiting vascular sprouting and branching. This leads to a lower number of capillaries and a poorly formed vascular network. Impaired angiogenesis in aged individuals is likely due to changes in the extracellular matrix. The extracellular matrix binds to blood vessel precursor cells to direct vascular sprouting and induce capillary formation. As individuals age, several extracellular matrix proteins change their form. Collagen fibrils become longer and thicker; elastin structure becomes disordered; and the globular structure of fibronectin is changed. Moreover, other extracellular matrix proteins such as decorin and laminin undergo changes in expression. The net result is a stiffer extracellular matrix which impedes angiogenesis (13). The trigger for these changes in the extracellular matrix is age-induced increases in the numbers of senescent cells (13).

### Regulation of fibrosis

Fibrosis is the development of scars due to excess deposition of extracellular matrix components in response to injury. In the absence of meaningful cardiac muscle regeneration, fibrosis maintains the integrity of the heart after injury. Myofibroblasts are key effectors; however, notable roles are also played by immune cells such as macrophages, mast cells, and lymphocytes (Fig. 1*B*).

Research into the mechanisms of fibrosis comes mostly from younger animals. Secreted molecules that induce fibrosis include proinflammatory cytokines (IL-1α, IL-1β, IL-6, TNF-α, and C-X-C motif ligands-1, 2, 5, and 8), mast cell-derived proteases, endothelin-1, and growth factors such as platelet derived growth hormone and transforming growth factor (TGF)-β (14, 15, 16). In addition, mechanical stressors promote fibrosis *via* integrins and mechanosensitive ion channels. Once stimulated, integrins and mechanosensitive ion channels induce fibroblasts to secrete collagens *via* the activation of a number of kinases including focal adhesion kinase, Rho-associated protein kinase, and mitogen-activated protein kinase (17). Due to its central role in mediating the differentiation of cardiac fibroblasts to myofibroblasts, TGF-β has been the main focus for the development of antifibrotic therapies (Fig. 1*B*) (18, 19). Whether such a strategy is viable in older individuals is less clear. In young individuals, cardiac fibroblasts respond robustly to TGF-β and the fibrotic scar that they form is effective in maintaining heart integrity. With aging, the cardiac fibroblast responses to TGF-β diminish. Reduced responses to TGF-β results in scars that are inadequate and the stresses that this imposes on the heart can lead to organ failure (20).

Aging is also associated with interstitial fibrosis, a broad, diffuse scarring that affects cardiac function. The extracellular matrix within the heart is maintained by cardiac fibroblasts. These cells secrete various matrix proteins as well as matrix-modifying enzymes. In young individuals, cardiac fibroblasts are effective in maintaining the extracellular matrix. In contrast, in aged individuals, cardiac fibroblasts no longer respond correctly to external stimuli. In their dysfunctional state, cardiac fibroblasts are unable to effectively manage collagen. Collagen fibers build up between cells and results in interstitial fibrosis. Interstitial fibrosis stiffens the heart walls, leading to systolic dysfunction and heart failure. Heart failure as a consequence of interstitial fibrosis is very common, and due to the aging population, the incidence is projected to double within 40 years. To date, there are no effective treatments. From a mechanistic viewpoint, aging associated increases in extracellular signal-regulated kinase (ERK)1/2 signaling have linked to interstitial fibrosis (20). While the exact reason for increased ERK1/2 signaling is unclear, increases in circulatory levels of growth factors such as insulin as well as age-related decreases in the expression of phosphatases such as DUSP1 and protein phosphatase 2A may play a role (20). Similarly, increased levels of reactive oxygen species(ROS) are a known trigger for interstitial fibrosis through the activation of ERK1/2 (21, 22, 23). Pertinent to the scope of this review, mice lacking the ROS scavenger superoxide dismutase quickly exhibit rapid onset of cardiac fibrosis and succumb within a short span of life, whereas overexpression of this enzyme prevents cardiac fibrosis in aged mice (24, 25).

### Cardiomyocyte generation *via* direct reprogramming

Injury and aging results in the permanent loss of cardiac muscle cells (cardiomyocytes). One option to regenerate the cardiomyocyte population is through direct cellular reprogramming. Direct cellular reprogramming involves the conversion of one somatic cell type into another without the need for a stem cell intermediate. In cardiac regeneration research, cardiomyocytes are generated from the direct reprogramming of fibroblasts. This has been achieved *via* pharmacological inhibitors, transcription factors and miRNAs (26, 27, 28, 29). Initially, the process was generally inefficient due to a lack of understanding of the mechanisms involved. By its very nature, reprogramming has to overcome potent barriers that safeguard cell identity. Thus, research in the field has concentrated on identifying these barriers. One such barrier is epigenetic modifications. Cardiomyocyte genes are silent in fibroblasts by virtue of inhibitory histone modifications such as H3K27me3 (30, 31) and H2AK119ub (32). These inhibitory histone modifications have to be actively maintained and this is achieved by Bmi1, Cbx1, PurB, and Sp3 (32, 33). The expression of these proteins is reduced by reprogramming factors, resulting in replacement of inhibitory histone modifications for those that promote transcription such as H3K4me3 (30, 31) (Fig. 1*C*). On their own, reduced expression of Bmi1, Cbx1, PurB, and Sp3 has little effect on fibroblast gene expression. This is a problem because continued expression of fibroblast genes would prevent the reprogramming cells from fully becoming cardiomyocytes. Fibroblast gene suppression can be achieved by inhibiting the TGF-β (34, 35, 36, 37) and Notch (38) signaling pathways. Both TGF-β and Notch are profibrotic, suggesting that profibrotic pathways represent a barrier to reprogramming. However, Akt1 activation, which is profibrotic, has been reported to be necessary for reprogramming (39) (Fig. 1*C*).

Another potential barrier to reprogramming is provided by autophagy. Autophagy is a crucial cellular process responsible for breaking down cytoplasmic proteins and organelles to recycle their components. Pharmacological activation of the autophagy mediator Atg5 was found to improve reprogramming efficacy (40). Surprisingly, the same was found when the authors depleted another autophagy mediator, Becn1. The authors concluded that Becn1 knockdown induced Wnt signaling and suggested that Becn1 was operating independently of its effects on autophagy (40). Due to the complex picture provided, further studies are warranted.

An additional barrier is the requirement to activate innate immunity pathways. It was initially observed that the method by which reprogramming factors were delivered into cells strongly affected outcomes; only those modes of delivery that induced innate immunity were found to be successful. Further studies into the mechanisms by which innate immunity regulated reprogramming identified important roles for the RNA-sensing receptors toll-like receptor (TLR) 3 and Rig1. TLR3 and Rig1 promote reprogramming through epigenetic pathways as well by activating transcription factors which stimulate cardiomyocyte gene expression (41, 42, 43) (Fig. 1*C*).

Studies in fibroblast to cardiomyocyte reprogramming have been conducted solely in neonatal and young animals. Thus, it is open to conjecture as to how the aforementioned barriers are affected by aging. Moreover, aging itself may also impose additional barriers. The latter appears quite likely as targeting the oxygen sensor Epas1 was found to improve fibroblast to cardiomyocyte reprogramming efficacy in older mice while having no effect in neonatal animals (44). Another potential issue is the extent to which reprogrammed cells retain aging-related features of the original cell type. Indeed, this has been observed in the direct reprogramming of fibroblasts into neurons (45). Carrying over aging-related fibroblast dysfunction into cardiomyocytes could potentially impair cardiac function.

### Cardiomyocyte proliferation

Adult cardiomyocytes proliferate weakly, if at all. Thus, cardiac regeneration could be achieved if these adult cardiomyocytes were coaxed to proliferate. The focus on adult cardiomyocyte proliferation has been considerable. Demonstrating true cardiomyocyte division has proven to be quite difficult as the cells will often express various cell cycle proteins, including those involved in cytokinesis, without actively dividing. Moreover, it is important not to just restore numbers but also functionality. Cardiomyocytes derived from inducible pluripotent stem cells (iPSCs) could potentially generate sufficient quantities for meaningful regeneration. However, to date, iPSC-derived cardiomyocytes are functionally immature.

Many growth factors have been reported to induce cardiomyocyte division in young animals. However, the majority only increases cardiomyocyte cell size and only a few appear to lead to cytokinesis. Growth factors associated with cytokinesis, and thus effective proliferation, include neuregulin-1, insulin-like growth factor-1, fibroblast growth factor-1, and TNF-related weak inducer of apoptosis (46, 47, 48, 49, 50) (Fig. 1*D*).

Growth factors mediate their effects on proliferation through signaling pathways. Signaling pathways identified as being important for cardiomyocyte division include PI3K-Akt, Wnt/β-catenin, and Hippo-YAP (51, 52, 53, 54). These pathways reinforce each other in a positive feedback loop, beginning with PI3K-Akt phosphorylation and inactivation of GSK3β. In the absence of GSK3β activity, β-catenin is stabilized and accumulates in the nucleus (55). Nuclear β-catenin is a necessary cofactor for YAP-mediated transcription (56). One of the targets of the YAP transcription factor is Pi3kcb (54). To close the loop, Pi3kcb activates PI3K-Akt (54) (Fig. 1*D*).

The PI3K-Akt, Wnt/β-catenin, and Hippo–YAP pathways promote division by modulating the expression of proteins involved in the cell cycle. The events of the cell cycle are governed by cyclins and their binding partners, the cyclin-dependent kinases (CDKs). Cyclins and CDKs mediating the effect of the aforementioned signaling pathways include cyclin-A2, cyclin-B1, cyclin-D1, cyclin-D2, CDK1, and CDK4 (57, 58, 59, 60) (Fig. 1*D*). PI3K-Akt, Wnt/β-catenin, and Hippo-YAP signaling also affect the expression of cell cycle inhibitors. CDK inhibitors block cell cycle progression by reducing CDK-cyclin activity. Adult cardiomyocytes express the CDK inhibitors p21, p27, and p57 (61). Under conditions that stimulate PI3K-Akt, p27 expression is reduced and cardiomyocytes undergo G2/M phase progression (62).

Manipulating these mechanisms is effective in restarting cardiomyocyte proliferation in young animals. Research suggests reduced efficacy in aged individuals. The capacity of cardiomyocytes to proliferate decreases with age (63, 64, 65, 66); implying that aging promotes proliferation resistance. The impact of proliferation resistance is further compounded by findings that suggest that activation of PI3K–Akt and Wnt/β–catenin pathways are deleterious in aged individuals. In aged mice, inhibition of PI3K-Akt signaling reduced age-related declines in cardiac function. The antiaging effects of reduced PI3K-Akt signaling activity were related to enhanced oxidative phosphorylation and increased autophagic flux (67). The authors of this study also noted enhanced PI3K-Akt signaling activity in failing human hearts (67). Similarly, aging is also associated with increased β-catenin levels (68) and this may play a role in pathophysiological remodeling (69). Human data with respect to aging and the activity of Hippo–YAP pathway is lacking. However, results from related systems are promising. To explain the promising data, a primer on Hippo-YAP signaling is needed. When the Hippo–YAP pathway is active, YAP is restrained in the cytoplasm. When the pathway is in the inactive state, YAP moves to the nucleus and activates genes. Interventions designed to turn the Hippo–YAP pathway off have been shown to induce cardiomyocyte proliferation in mice up to 8 months of age, equivalent to early middle age in humans (52). Moreover, heart failure in humans has been shown to activate Hippo-YAP (70).

## What can aging research teach us about cardiac regeneration?

As described in the previous section, the cardiac regenerative field has identified many mechanisms that effectively regenerate the hearts of young, but not aged, individuals. Interventions based on these mechanisms have not arrived in the clinic. This may be because they have not fully taken into account the effects of aging. In the previous section, we discussed how the main mechanisms are affected by aging. However, there is plenty of additional aging research that could be used to help cardiac regeneration. That research is the focus of this section.

### Reactive oxygen species

Cellular homeostasis relies on the oxidation-reduction (redox) system. Under physiologic conditions, the redox system maintains a balance between the generation and removal of ROS. A short duration of increased ROS may limit injury. However, if increased ROS levels are prolonged then significant damage occurs to cells. The importance of ROS in cardiac aging has been shown through the overexpression of antioxidant proteins. Expression of the antioxidant enzyme catalase in mitochondria improves lifespan in mice and reduces cardiac aging phenotypes such as hypertrophy and diastolic dysfunction (71). Aging leads to changes in the expression of enzymes involved in maintaining the equilibrium of the redox system, tipping the balance toward increased ROS levels. Exogenous sources of ROS include nitric oxide oxidase and NADPH oxidases. However, the dominant source of ROS in aging is the mitochondria (72).

**Figure 2:**
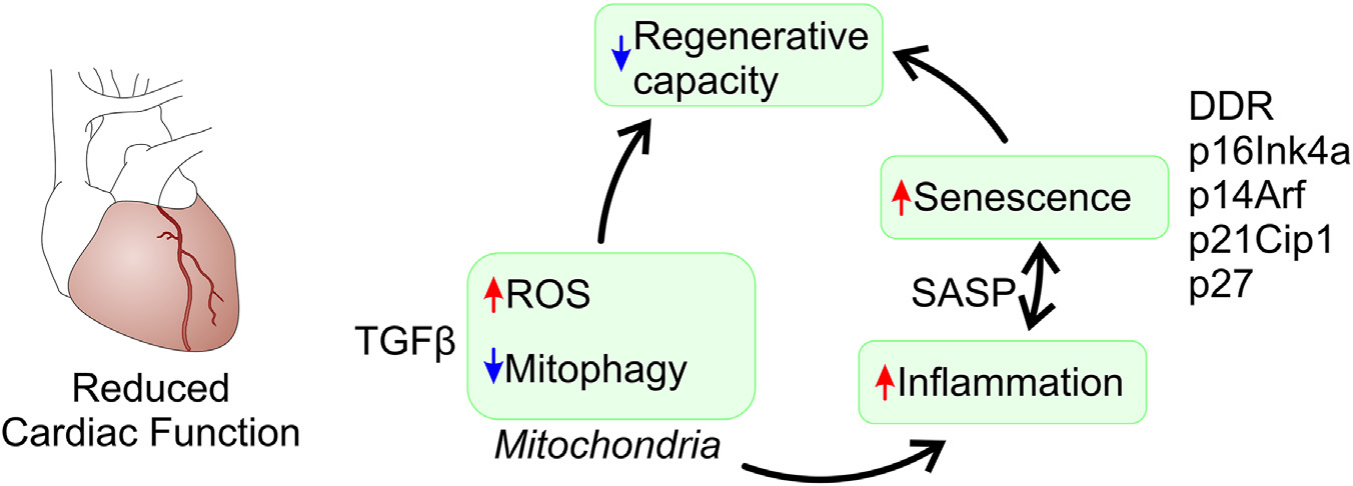
**Molecular pathways in cardiac aging.** Aging is associated with reduced cardiac function. As the heart ages, mitochondria become dysfunctional and cells become senescent. The net result is an increase in ROS levels and the production of proinflammatory proteins respectively. ROS and proinflammatory proteins set up a positive feedback loop which increases cellular senescence and impairs regenerative capacity. ROS, reactive oxygen species.

Mitochondria generate ATP through the electron transport chain. The electron chain is comprised of four enzymatic complexes numbered I, II, III, and IV. Complexes I and III are the main sources of ROS. Aging induces a defect in the ubiquinol binding site (Q(O)) within complex III, the result being increased ROS production. Increased ROS generation from complex III disrupts calcium handling within the endoplasmic reticulum, inducing stress within the organelle. A stressed endoplasmic reticulum impairs complex I activity, which also increases ROS generation. Increased ROS generation weakens mitochondria. Mitochondrial integrity is maintained in part by the phospholipid cardiolipin. In response to increased ROS, cardiolipin levels fall, resulting in damage to the inner mitochondrial membrane. Similarly, increased ROS also promotes damage to the mitochondrial genome (Fig. 2) (72).

Increased ROS production, structural changes, and genetic damage are the hallmarks of a dysfunctional mitochondria. The process by which dysfunctional mitochondria are removed is called mitochondrial autophagy (mitophagy). In young individuals, mitophagy is effective and dysfunctional mitochondria are rapidly cleared before excessive ROS production can do significant damage. Mitophagy becomes less effective in the aging heart, leading to ever increasing numbers of dysfunctional mitochondria and a vicious circle of ever increasing ROS generation (Fig. 2) (73, 74).

Increased ROS not only damages mitochondria but it also promotes interstitial fibrosis, hypertrophy, senescence, inflammation, and contractile dysfunction.

ROS appears to combine with cytokines and angiotensin-II to promote interstitial fibrosis through TGF-β (75). Exacerbating the situation, angiotensin-II levels in the blood increase with age. Levels of angiotensin-II in the circulation are regulated by the renin-angiotensin-aldosterone system and renin-angiotensin-aldosterone system inhibitors such as angiotensin-converting enzyme inhibitors or AT1 receptor antagonists reduce age-related interstitial fibrosis (75).

There are a number of mechanisms by which ROS can induce cardiomyocyte hypertrophy. In addition to promoting interstitial fibrosis, ROS and angiotensin-II also combine to induce hypertrophy through the activation of PKC, c-Jun N-terminal kinase, and ERK. Similarly, ROS potentially induces hypertrophy by potentiating TNF-α signaling to matrix metalloproteinases. ROS also promotes hypertrophy by activating the Na+/Ca2+ and Na+/H+ exchangers NCX and NHE1, respectively. These exchangers function *via* a Ca2+-dependent pathway. As a side effect, elevated Na+ may also explain the increased incidence of arrhythmia that is seen with advancing age (76).

Cells in the mammalian heart exit the cell cycle after birth and gradually becoming senescent. These cells accumulate in the heart where they promote organ dysfunction and decreased lifespan (77). Despite being unable to replicate, senescent cells remain metabolically active and they are marked by the so-called senescence-associated secretory phenotype (SASP). The SASP is a collection of proinflammatory proteins, proteases, and extracellular matrix proteins. Through paracrine effects, SASP proteins impair the function of adjacent cells (78). Similar to how ROS self-amplifies, SASP signaling propagates cellular senescence by inducing p53 and CDK inhibitors such as p16INK4A, p14ARF, p21CIP1, and p27 in neighboring cells (79, 80, 81). The wave of increasing numbers of senescent cells is also amplified by declines in the efficiency of mechanisms to remove them (82) (Fig. 2). Mitochondrial dysfunction may also act as a trigger for age-related inflammation. Mitochondrial DNA fragments activate TLR9 signaling, triggering the release of proinflammatory cytokines and subsequent inflammatory reactions within cardiomyocytes (83).

Clinically, pathological oxidative stress is combated *via* exogenous antioxidants. Vitamins A, C, and E are commonly employed and reduce ROS by increasing antioxidant enzyme activity, promoting scavenging and preventing fat oxidation, respectively (2). Other agents that can potentially reduce ROS include zinc and selenium, as well as carotinoids and flavonoids (2). Interestingly, vitamin C and selenium also appear to be important for cardiomyocyte differentiation (84, 85), suggesting that combining anti-ROS agents with cardiomyocyte proliferation methods could be beneficial in aged individuals.

Stem cells injected into the heart reverse various aging effects in the heart, including reversing age-associated ROS production. This occurs through paracrine mechanisms, whereby the injected stem cells induce GSH transferase and superoxide dismutase activity in adjacent cells (86). Research into stem cell injections has stagnated for various reasons. This is unfortunate because paracrine factors could be a “one-stop shop” as they have been shown to reduce many aging effects as well as being proregenerative (87). Exosomes are believed to be important carriers of paracrine factors and are a viable alternative to cell-based therapies (88). Similarly, bioengineering is also a viable option for the application of stem cells (89).

Elevated ROS production is not the only consequence of dysfunctional mitochondria. With aging, mitochondria also become less efficient at producing ATP. Reduced efficiency in producing ATP is due to a decrease in oxidative phosphorylation. Similarly, with age, calcium homeostasis also becomes dysregulated due to calcium mishandling and cytosolic calcium overload. This is caused in part by reduced expression of the mitochondrial calcium uniporter and MICU1. Triggers for mitochondrial dysfunction include endocrine dysfunction, systemic inflammation, nutrition, and the environment. While it is outside the scope of this review to discuss the above at length, the interested reader is directed to recent reviews on the topic (90, 91, 92). However, it is important to note that reduced ATP production, a breakdown in calcium homeostasis, endocrine dysfunction, etc are all potentially relevant for cardiac regeneration.

### Genetic damage and telomere loss

Telomeres, repetitive DNA sequences found at the ends of chromosomes, play a crucial role in maintaining genome integrity (93). With each cell division, telomeres shorten and beyond a certain point DNA is damaged (94). Although cardiomyocytes are predominantly postmitotic cells, telomere shortening is still relevant in the context of cardiac aging. Telomerase deficiency, which leads to severely shortened telomeres, has been linked to age-related cardiac dysfunction and myocardial remodeling (95). Dysfunctional telomeres activate DNA-damage response proteins and in so doing, promote cellular senescence through a phenomenon known as telomere-associated DNA damage foci (78, 96, 97) (Fig. 2). Telomere-associated DNA damage foci trigger classic senescence pathways involving p21CIP and p16INK4a.

### Reversing the effects of aging through partial reprogramming

Partial reprogramming is a strategy to return cells to a younger phenotype. In short, partial reprogramming utilizes transient expression of pluripotency-associated reprogramming factors, such as the Yamanaka factors Oct4, Sox2, Klf4, and c-Myc (OSKM). Transient expression of OSKM induces a youthful state while avoiding full reprogramming to an iPSC state (98, 99). Partial reprogramming has been shown to erase certain epigenetic marks of aging, effectively “rewinding” the cellular clock while preserving the cell's identity. Researchers have discovered that partial reprogramming could potentially enhance the regenerative potential of cardiomyocytes, possibly leading to improved functionality and longevity of heart tissue (100). Cardiac-specific expression of OKSM induced adult cardiomyocytes to dedifferentiate and reenter the cell cycle. The cardiomyocytes more closely resembled their fetal counterparts. Short-term OKSM expression was important as prolonged OKSM expression led to cellular reprogramming and the formation of tumors in the heart (100).

## Perspectives

Despite a prolonged effort and promising results in animal studies, not a single cardiac regenerative strategy has reached the clinic. The position of this review is that this is result of a disconnect between the youth of research animals and the age of the patients that need to be treated.

### Aging influences the effectiveness of current cardiac regenerative strategies

Cardiac regenerative strategies typically resolve around revascularization, resolving fibrosis or cardiomyocyte generation. To summarize the earlier discussion, these strategies are impacted by age. Aging reduces the capacity for revascularization. Thus, revascularization in an aged population *via* the proposed methods is likely to be less effective or require higher stimulant doses. The latter potentially runs into issues of cost and off-target effects. Similarly, aged fibroblasts respond differently to fibrotic stimuli. Injury-induced fibrotic scars are weaker in aged individuals. Moreover, interstitial fibrosis, fibrosis in the absence of injury, becomes apparent. These effects point to significant phenotypic change in fibroblasts. Considering that young and aged fibroblasts are apparently phenotypically distinct, it is not surprising that mechanisms identified as being antifibrotic in the young are not effective in the aged individual. Cardiomyocyte generation has been achieved either by cardiomyocyte proliferation or through the reprogramming of scar fibroblasts. The former has been muddied by studies reporting increased expression of one or more proliferation markers without demonstrating true cytokinesis. True cytokinesis can be achieved in cardiomyocytes in young research animals *via* activation of the PI3K–Akt, Wnt/β–catenin, and Hippo–YAP pathways. Nevertheless, these pathways become dysregulated with increasing age. Reprogramming of fibroblast to cardiomyocytes has not been studied in aged animals. This has been a strategy of convenience as reprogramming efficacy is essentially nonexistent in cultured adult fibroblasts (44). Recent research suggests that the focus on reprogramming in very young animals is missing important aging induced barriers. For one, as mentioned above, fibroblasts change phenotype with age. By early adulthood, cardiac fibroblasts begin to express the oxygen sensor Epas1 and that this protein is a barrier to reprogramming (44). It also appears that reprogrammed cells retain aging-related features of the original cell type (45). Thus, aging-related fibroblast dysfunction could manifest itself *via* the generation of dysfunctional cardiomyocytes.

The problem with ignoring the effects of aging can be highlighted by the cautionary tale of anti-inflammatories. Following cardiac injury, there is a strong inflammatory response in young research animals. In these individuals, dampening inflammatory responses *via* anti-inflammatories reduced injury-associated cardiac damage. Based on this research, anti-inflammatories were used in several clinical trials in an attempt to limit infarct damage. All of these trials failed to show any efficacy and the anti-inflammatories were found to have no effect on acute cardiac damage or remodeling (101). While the initial work on anti-inflammatories was carried out in young individuals, the clinical trials were performed in aged population where acute inflammatory responses are much weaker, suggesting a flawed design. When age effects are taken into account, clinical trials are more effective. Baseline expression of inflammatory proteins increases with age and trial data reporting the effects of targeting the proinflammatory protein IL-1β in a nonacute setting stated a reduced reoccurrence of adverse cardiac advents (102).

### How aging research can help cardiac regeneration

As described in section 4, there is abundant research into the effects of aging. However, the findings of aging research receive scant attention in the field of cardiac regeneration. This is particularly evident with respect to mitochondrial dysfunction and elevated ROS, as well for senescence. Mitochondrial dysfunction and ROS appear to be good targets for cardiac regeneration in aged individuals. Indeed, delivery of coenzyme Q10 *via* the MITO-Porter system improves mitochondrial dysfunction (103). In addition, there are several other strategies targeting ROS production specifically in the mitochondria. These include MitoTEMPO, MitoQ, and SS-31/MPT-131. MitoTEMPO is a mitochondrial binding form of piperidine nitroxide. Piperidine nitroxides reduce ROS due to their ability to cycle between nitroxide, hydroxylamine, and oxoammonium forms. In animal models of heart failure, MitoTEMPO reduced ROS levels and reversed cardiac remodeling as well as improving mitochondrial function and heart contractility. MitoQ is a mitochondrial matrix-targeted ubiquinone. Once in the matrix, ubiquinone reduction to ubiquinol reduces ROS levels by reducing lipid peroxidation. Like MitoTEMPO, MitoQ improves mitochondrial function and heart contractility in injury models. SS-31 belongs to the group of Szeto-Schiller (SS) peptides, which are known to selectively bind to the inner mitochondrial membrane. The target for SS-31 is cardiolipin. By binding to cardiolipin, SS-31 prevents it from converting cytochrome c from an electron carrier into a peroxidase. As a result, SS-31 promotes the electron transport chain at the expense of ROS production. In several small and large animal models, SS-31 prevents hypertrophy, reduces fibrosis and improves cardiac function. Due to these results, several clinical trials of SS-31 in its acetate salt variant (MTP-131/bendavia/elamipretide) have been conducted. In a trial of patients with heart failure with preserved ejection fraction, MTP-131 improved cardiac function. However, in patients with ST-elevated myocardial infarction, there was no significant impact on infarct size or ventricular function (104). Beyond mitochondrial dysfunction and ROS, another attractive target for cardiac regeneration in aged individuals is senescent cells. Drugs that specifically induce senescent cells to undergo apoptosis are called senolytics (105, 106, 107). Preclinical trials suggest that senolytics can revert some of the effects of aging, such as improving vascular reactivity and cardiac function (108, 109). Beyond senolytics, there are a number of emergent technologies that specifically target senescent cells. Proteolysis-targeting chimeras degrade harmful proteins within senescent cells (110). In addition, galactose-based prodrugs exploit the heightened lysosomal activity in senescent cells, allowing for the selective release of cytotoxic agents within these cells and thereby sparing nonsenescent cells (111, 112). Finally, enhancing the immune system's natural capacity to clear senescent cells has been achieved through chimeric antigen receptor *T*-cell therapy (113). With the ready availability of ROS scavengers, senolytics, and the like, there are plenty of tools to investigate how to achieve cardiac regeneration in the aged population.

### Combination therapies

An important way that ROS scavengers, senolytics, and the like could be helpful for cardiac regeneration in the aged population is through combination therapies. Combination therapies can be employed to target several processes in the same cell. The study of Waypa *et al.* suggests one possible route. This study is notable because it links cardiomyocyte proliferation with decreased mitochondrial function (114). Deletion of the mitochondrial Rieske iron–sulfur protein (Uqcrfs1) caused a decrease in mitochondrial function and metabolic shift. This shift in metabolism was associated with an increase in glucose utilization and SAM levels, while levels of alpha-ketoglutarate decreased. Concomitant with this metabolic shift, cardiomyocyte numbers were doubled (114). As mentioned earlier, dysfunctional mitochondria are a hallmark of aging. These dysfunctional mitochondria are less efficient; however, they are also super producers of ROS. In the Waypa *et al.* study, ROS generation was reduced in the Rieske iron–sulfur protein knockout. Taken together, this suggests that if ROS overproduction was targeted it could be possible to co-opt the reduced efficiency of aged mitochondria to drive effective cardiomyocyte proliferation.

The alternative to targeting multiple processes in a single cell would be to target several cell types. One such example would be to combine cardiomyocyte proliferation with revascularization. The expectation is that targeting several cell types would provide synergistic benefits (115). Indeed, it has been shown that human iPSC-derived endothelial cells improve the maturity and function of human iPSC-derived cardiomyocytes. When coinjected into nonhuman primates after ischemic reperfusion, significantly enhanced vasculature and improved cardiac function was observed (116). It is also important to note that while the field tends to focus on cardiomyocytes, fibroblasts, and endothelial cells, there are other cell types in the heart that potentially play a role in regeneration (117).

## Conclusion

In conclusion, cardiac regenerative strategies are investigated predominantly in young adult animals. However, there are significant differences in the young adult and aged adult heart both chronically and in how they deal with injury. These differences are likely to stymie the progression of cardiac regeneration and may explain why no cardiac regenerative strategy has reached the clinic. The premise of this review is that cardiac regenerative strategies need to take aging into account. Some work has been done, notably in fibrosis and oxidative stress. Nevertheless, the impacts of many aspects of aging, such as mitochondrial dysfunction, reduced innervation, abnormal calcium homeostasis, and decreased capillary density, on cardiac regeneration are still unknown.

## Conflict of interest

The authors declare that they have no conflicts of interest with the contents of this article.
